# Gender differences in dentistry and oral sciences research productivity by researchers in Nigeria

**DOI:** 10.3389/froh.2023.1059023

**Published:** 2023-04-27

**Authors:** Morenike Oluwatoyin Folayan, Maha El Tantawi, Erfan Shamsoddin, Guillermo Z. Martínez-Pérez

**Affiliations:** ^1^ Faculty of Health Sciences, University of Zaragoza, Zaragoza, Spain; ^2^ Department of Child Dental Health, Obafemi Awolowo University, Ile-Ife, Nigeria; ^3^ Department of Clinical Sciences, Nigeria Institute of Medical Research, Yaba, Nigeria; ^4^ Community Oral Health Department, Tehran University of Medical Sciences, Tehran, Iran; ^5^ Department of Pediatric Dentistry and Dental Public Health, Alexandria University, Alexandria, Egypt; ^6^ Cochrane Iran Associate Centre, National Institute for Medical Research Development, Tehran, Iran

**Keywords:** open access publishing, author position, collaboration pattern, total citations, papers published, dentistry and oral sciences research

## Abstract

**Background:**

The aim of the study was to assess gender differences in the productivity, impact, collaboration pattern and author position of dentistry and oral sciences researchers in Nigeria.

**Methods:**

We examined the Web of Science (WoS) publication records of dentistry and oral sciences researchers to assess gender differences in productivity, impact, collaboration and authorship pattern (first authorship, last authorship and corresponding author). The analysis included the number of publications in journals ranked based on their quartile rating amongst the journals in the subject area (Q1–Q4). Chi square was used to make gender comparisons. Significance was set at >5%.

**Results:**

413 unique authors published 1,222 articles on dentistry and oral sciences between 2012 and 2021. The number of WoS documents per female author was significantly higher than that per male author (3.7 vs. 2.6, *p* = 0.03). A non-significantly higher percentage of females authored papers in Q2 and Q3 journals and a higher percentage of males authored papers in Q4 journals. The number of citations per female author (25.0 vs. 14.9, *p* = 0.04) and the percentage of females listed as first authors (26.6% vs. 20.5%, *p* = 0.048) were statistically greater than men. The percentage of males listed as last authors was statistically greater than females (23.6% vs. 17.7%, *p *= 0.04). The correlation between the percentage of papers with researchers listed as first authors and that listed as last authors was not significant for males (*p* = 0.06) but was significant for females (*p* = 0.002). A non-significantly greater percentage of females were listed as corresponding authors (26.4% vs. 20.6%) and males were listed as international (27.4% vs. 25.1%) and domestic collaborators (46.8% vs. 44.7%). Also, there was no statistically significant gender difference in the proportion of articles published in open access journals (52.5% vs. 52.0%).

**Conclusion:**

Though there were significant gender differences in the productivity, impact, and collaboration profile of dentistry and oral sciences researchers in Nigeria, the higher female research productivity and impact may be driven by cultural gender nuances that needs to be explored further.

## Introduction

Research productivity is a topic of interest for researchers. This is because scientific research productivity is linked to the intellectual wealth and economic progress of countries (1, 2). Conversely, the intellectual wealth and economic progress of countries is linked to the physical and psychosocial health and well-being of its citizens (3) which is driven by research (4). This nexus is not surprising since the primary objective of conducting research is to explore answers to questions that have social value (5). This nexus of inter-dependency of economic growth, health and research makes it increasingly important for academic and research regulatory systems to study and understand how biomedical, socio-epidemiological and clinical research performance in the university system could be improved (6).

Research performance can be measured through academic publication. The dissemination of academic publication is a proof of academic faculty members' performance and achievement, and an indicator of excellence for universities (7). The faculty member's academic performance is measured by the number of published articles in indexed databases (8, 9). One factor that affects research productivity is gender. Gender gaps in academia are well documented in industrialized and developing economies. These gender gaps include the inequity in earning grants and awards, participation in the scientific workforce, holding of senior and leadership positions and publication and citation rates (10–14). There are multiple evidences that men publish and are cited more often than women irrespective of the field of research (15). This is called the “Matilda Effect” (16).

Reasons for the “Matilda Effect” range from gender differences in family responsibilities (17) to more female dedicated time to serve on committees, teaching and mentoring students (18, 19), gender bias in peer review (20) and unequal resource allocation to male and female researchers (21). Also, females publish significantly fewer papers in research areas that require huge funding (21) and are less likely to participate in collaborations that lead to publications (22). They are also less likely to be listed as either the first or last author on a published article (22), and receive about 10% fewer publication points per publication than men (23). This disparity persists among elite scientists, including those in Africa (24).

In Nigeria, - there was a 60% increase in research publications between 2008 and 2017 (25, 26), and the average number of publications by women was more than that by men (10.8 vs. 9.7) (27). Research publications in Nigeria were heavily skewed towards the environmental, health, public and occupation domains (27), like Agriculture, Veterinary, Immunology and Medicine disciplines (1). This skewness aligns with the country's need for food security and infectious disease management (28). This is unlike the similarities in the relative importance of different research disciplines and their contributions to economic development in high-income countries (1).

An area of biomedical research in its infancy in Nigeria is dentistry. The academic pursuit in dentistry and oral sciences only started in 1965 with the establishment of the School of Dentistry, University of Lagos. Dentistry and oral sciences is an important discipline as the human and economic development of a country linearly correlates with dental research productivity (29). Oral health research advances the health of the population (30). It may however, be assumed that just like in the period of infancy of the medical and like in high-income countries, research productivity in dentistry may be favorably skewed towards men (30). An analysis of the gender distribution of publications in the field of dentistry and oral sciences in Nigeria, and the factors that influence the distribution will help support the establishment of gender supportive schools of Dentistry in the West Africa sub-region and other countries with profiles similar to Nigeria.

Our theoretical assumptions for this study were based on the academic literacies theory that treats reading and writing as social practices that vary with context, culture and genre (31); and the academia as a place where power is distributed unequally (32, 33). We conceptualized research productivity as the extent to which a researcher produces publications aimed at an academic audience (26). We assessed productivity using bibliometric measures that credit and count publications in the same manner regardless of the author's gender, but recognized that cultural nuances that promote gender inequality may also be reflected in the productivity of females when compared to males. Cultural nuances such as ethnicity, class and ability, influences how gender roles are proscribed in academia, with females being more engaged in academic housekeeping affairs and taking on low-prestige endeavours (34–38).

This study explored gender disparity in research productivity in dental science research in Nigeria. The aim was to assessing gender differences in the research productivity of dentistry and oral sciences researchers in Nigeria. The focus was on gender differences in research publications measured by productivity, impact, collaboration pattern, open access publishing and authorship pattern. The finding will guide the design and implementation of our next phase of research which is the qualitative explorations of the “why” and “how” the systems in the academia enables gender inequity in dentistry and oral sciences research productivity in Nigeria.

## Materials and Methods

This was a bibliometric review of 1,222 articles produced by 413 individuals and published over the 10-year period preceding this analysis (2012–2021). The bibliometric review was conducted in June 2022 and the study data were obtained from the WoS InCites electronic database. The WoS InCites electronic database was used because of its global recognition as a credible and comprehensive database for bibliometric analyses (39, 40).

We conducted an analysis in the WoS InCites dataset using the Web of Science schema for Research Area (Dentistry, Oral Surgery & Medicine for this study), applying the following filters: time period from 2012 to 2021, location (Nigeria) and document type (Article). The analysis excluded documents like book chapters, meeting abstracts, proceedings paper, meeting summary and others.

Data extraction were performed in three phases. The first phase was conducted by MET who searched the database for articles and downloaded the results as comma separated values (CSV) file. The results were then screened to ensure all required data were available. In the second phase, MET and MOF reviewed the titles and abstracts of the retrieved articles for suitability to ensure they met the inclusion criteria. In cases where there was conflict in the selection of an original article, the conflict was resolved through consensus building between the two authors. In the fourth phase, results were shared with ES for his review. Publications were retained when there was consensus between the three reviewers. The following information was extracted: authors' information (names and identity, document title, year of publication, journal title, volume, issue and page numbers, and citation count); bibliographical information (affiliations, serial identifiers of journal, language of original document, and journal publisher); and author keywords.

Authorship consists of a person and a paper for which the person is designated as a co-author (24). We included all authors listed in the Web of Science (WoS) InCites database for articles in the research area Dentistry, Oral surgery and Medicine affiliated with Nigerian institutions. This was possible as WoS InCites database classified all publications by field and enabled categorization of publications using citation information. This database is a digital archive of published scholarly research that spans the life sciences, biomedical sciences, engineering, social sciences, arts and humanities from 1900 to the present day (41). At the time of this analysis, the WoS InCites database had over 82 million articles, reviews, editorials, chronologies, abstracts, proceedings (journals and book-based) and technical papers in 256 disciplines. We focused on articles as type of publication because articles are used in university ranking systems (42). The articles in the WoS InCites database are derived from over 21,894 journals, 126,000 books and 226,000 conferences proceedings (43)].

**Figure 1:**
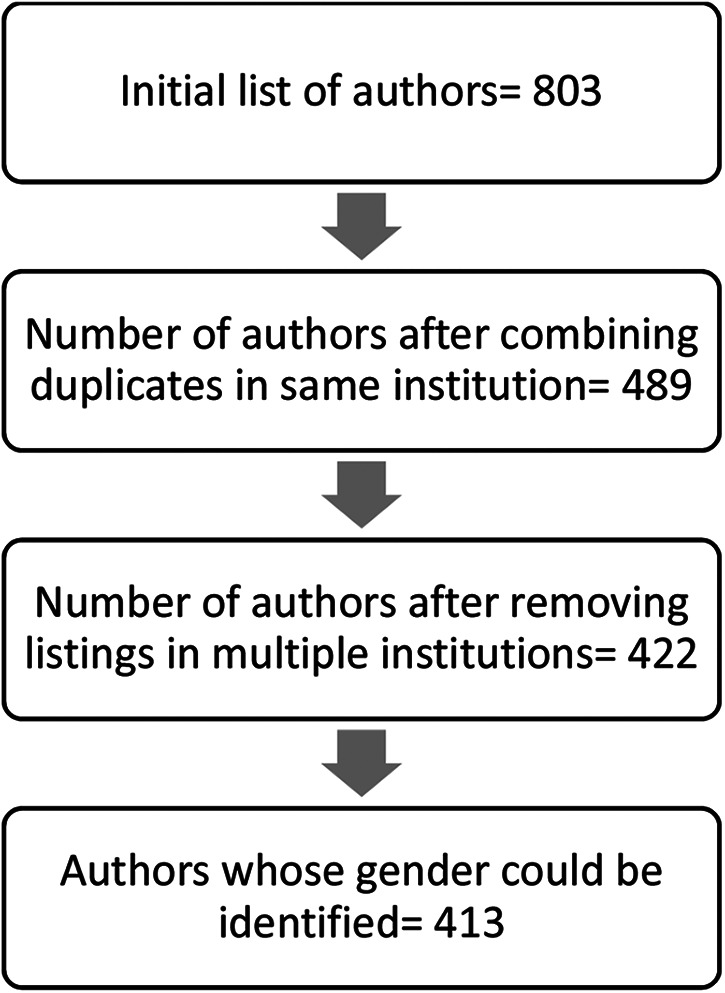
Number of authors identified at different stages.

Figure 1 is the flowchart of how we searched and identified the authors who published in Dentistry, Oral surgery and Oral medicine. When the name of an author was repeated in the same institution, we combined the counts of articles and averaged the category normalized citation impact (CNCI). When the author's name was repeated in more than one institution, we combined the counts and averaged the CNCI under the more recent affiliation identified through personal communication with heads of institutions or delegated key people. The names of some authors affiliated with the University of Ibadan were repeated with the University of Ibadan Teaching Hospital affiliation. We removed the later and kept only the affiliation of the university since the teaching hospital is a subset of the university.

We identified the sex of the authors based on one of the authors' (MOF) knowledge of some individuals who are colleagues. Also, the cultural and religious gendered connotations of the listed first name (22, 44) were used to ascribe gender with the assumption that that name is associated with a single sex (such as “Joseph”, “Mustapha” or “Babatunde” for male and “Victoria”, “Shekeerah” or “Yetunde” for female). We further corroborated the assigned sex by searching the web for pictures using the listed names and the institution address. We also personally contacted the heads of institutions and key institutional focal persons to identify the sex of listed individuals whose institutional contact addresses were written in the article. Where authors were affiliated with two institutions, we contacted the heads for both institutions to ascertain the workplace identification of the individual. We also used the institutional identification process to validate some of the individuals whom we have identified based on cultural and religious gendered connotations of the listed author's first name and we had 100% correctness in the sex assignment. We then determined the proportion of female authorships as the quotient between the number of female authorships and the total sum of male and female authorships presented as percentage.

We categorized the manuscripts published based on the ranking of the journals where the manuscript is published as indicated in the WoC InCites database. The journals ranking was based on their quartile rating amongst the journals in a subject area (Q1–Q4). Q1 journals are amongst the top 25% of a subject area, while Q4 journals are among the last 25% of a subject area. It also covered authorship (first and last-authorship as well as corresponding author). Single authorships were considered as first authorships.

We used an observation period of 10 years to provide larger and more robust datasets for each person. Data about the indicators of productivity, impact, collaboration pattern, open access publishing and author position were extracted and highlighted in Table 1 (45, 46).

**Table 1:** Definition of research indicators used in the study.

Indicator	Definition
**Productivity**	
WoS documents	Number of publications published in journals in WoS
Percentage in Q1 journals	(Count of documents in Q1 journals / count of documents in journals with impact factor)*100
Percentage in Q2 journals	(Count of documents in Q2 journals / count of documents in journals with impact factor)*100
Percentage in Q3 journals	(Count of documents in Q3 journals / count of documents in journals with impact factor)*100
Percentage in Q4 journals	(Count of documents in Q4 journals / count of documents in journals with impact factor)*100
**Impact**	
Times cited	Number of times the set of articles has been cited
Category normalized citation impact (CNCI)	Number of citing items divided by the expected citation rate for articles of the same document type, year of publication and subject area. It is a valuable and unbiased indicator of impact irrespective of age, subject focus, or document type. It allows comparisons between entities of different sizes and different subject mixes. A value of 1 represents performance at par with world average and values above 1 are considered above average and so on.
Percentage cited	Percentage of articles with at least one citation. It shows the extent to which other researchers utilize the research produced by an entity.
**Collaboration**	
Percentage international collaboration	Number of documents with international collaborations divided by the total number of documents represented as a percentage. It is an indication of ability to attract international collaborations
Percentage domestic collaboration	Number of domestic collaborations divided by the total number of documents represented as a percentage
**Open access**	
Percentage open access	Percentage of articles that are published using open access model including gold, hybrid gold, bronze, free to read, green published, green submitted, green accepted, and all green only.
**Author position**	
First author	The number of publications where the location is the location associated with its first author.
Last author	The number of publications where the location is the location associated with its last author.
Corresponding author	The number of publications where the location is the location associated with its reprint or corresponding author.

Based on articles, in the research area “dentistry, oral surgery and medicine”, in the WoS core collection, in the period 2012–21.

Chi-square was used to compare gender differences in the percentages of publications in Q1–Q4 journals. Also, the gender differences in percentage cited, percentage of papers with international collaboration, percentage with domestic collaboration, percentage of publications in open access journal and percentage with first, last and corresponding author were compared using chi-square test. The number of WoS documents, number of citations, and CNCI were compared using t test. The percentage of papers with authors listed as first authors and those where they were listed as last authors were correlated using Pearson correlation coefficient after splitting the sample by gender. Significance was set at <5%. SPSS version 23.0 was used for statistical analysis.

## Results

Table 2 shows the analysis of the 1,222 articles authored by researchers affiliated with Nigerian institutions indexed in the WoS database. There was an average of three papers per author and a greater percentage in Q4 than in Q3, Q2 and Q1 journals (22.1%, 15.6%, 14.2% and 10.7%). Most (77.3%) papers were cited with about 18.6 citations per author although the CNCI (0.60) was below the global average of 1. A greater percentage of papers listed domestic (45.8%) than international (26.4%) collaborators, and 52.2% of the articles published in open access journals. Less than one in four papers had authors listed as first (23.2%), last (20.9%) or corresponding authors (23.2%).

**Table 2:** Comparison between male and female authors affiliated with Nigerian institutions regarding research productivity, impact, collaboration patterns, open access publishing and authorship patterns in dentistry and oral sciences.

Variables	Combined papers in the study			*p*-value
	All papers	By females	By males	
**Productivity**				
Number of Web of Science document (per author)^¶^	1,222 (3.0)	553 (3.7)	669 (2.6)	0.03
Percentage in Q1 journals	131 (10.7%)	59 (10.7%)	72 (10.8%)	0.96
Percentage in Q2 journals	174 (14.2)	87 (15.7%)	87 (13.0%)	0.24
Percentage in Q3 journals	191 (15.6%)	94 (17.0%)	97 (14.5%)	0.31
Percentage in Q4 journals	270 (22.1%)	113 (20.4%)	157 (23.5%)	0.31
**Impact**				
N citations (per author)^¶^	7,671 (18.6)	3,779 (25.0)	3,892 (14.9)	0.04
CNCI^¶^	0.60	0.59	0.61	0.84
Percentage cited	77.3%	79.0%	75.8%	0.63
**Collaboration**				
Percentage with international collaboration	26.4%	25.1%	27.4%	0.50
Percentage with domestic collaboration	45.8%	44.7%	46.8%	0.65
Percentage published in open access journal	52.2%	52.0%	52.5%	0.91
**Author position**				
Percentage first author	23.2%	26.6%	20.5%	0.048
Percentage last author	20.9%	17.7%	23.6%	0.04
Percentage corresponding author	23.2%	26.4%	20.6%	0.06

^¶^ : *t* test used for comparison and *χ*^2^ test used for all other comparisons.

Table 2 shows that 669 (54.7%) articles were authored by males and 553 (45.3%) were authored by females. The total number of citations of articles authored by males was higher than that for articles authored by females (3,892 vs. 3,779).

The number of WoS documents per female author was significantly higher than the number authored per male author (3.7 vs. 2.6, *p* = 0.03). The number of citations per female author was significantly higher than the number of citations per male author (25.0 vs. 14.9, *p* = 0.04). A non-statistically significant higher percentage of females authored articles in Q2 and Q3 journals, a non-statistically significant higher percentage of males authored articles published in Q4 journals, and males had a non-statistically significant higher CNCI (0.61 vs. 0.59) and a non-statistically significant lower percentage of articles cited (75.8% vs. 79.0%) than females.

In addition, although a greater percentage of articles published by male than female authors listed international collaborators (27.4% vs. 25.1%) and domestic collaborators (46.8% vs. 44.7%), none of these differences were statistically significant. Also, there was no statistically significant gender difference in the proportion of articles published in open access journals (52.5% vs. 52.0%, *p *= 0.91). However, there was a statistically significantly greater percentage of females than males listed as first authors (26.6% vs. 20.5%, *p* = 0.048) and a statistically significantly greater percentage of males than females were listed as last authors (23.6% vs. 17.7%, *p* = 0.04). The correlation between the percentage of articles with researchers listed as first authors and the percentage listed as last authors was not significant among males (Pearson correlation = 0.12, *p* = 0.06) but was significant among females (Pearson correlation = 0.25, *p* = 0.002). A greater percentage of females than males were listed as corresponding authors although the difference was not statistically significant (26.4% vs. 20.6%, *p* = 0.06).

## Discussion

The findings of the current study suggest that although the number of male authors from Nigeria publishing articles in the WoS category of dentistry and oral sciences was greater than the number of female authors, the quality of the published manuscripts by females seems to be higher than that by males judging by the significantly higher number of citations. The slightly higher percentage of articles authored by males listing international and domestic collaborators and their significantly higher listing as last authors suggests that male authors may initiate and engage more in mentorship, networking and partnership building. The significantly higher percentage of females listed as first authors observed may suggest female dental researchers in Nigeria play more junior roles.

The study produced a specialty-focused assessment of research by gender for a lower middle-income country. It is one of the few publications on the productivity and impact of oral health researchers in a low-middle -income country and, to the best of our knowledge, the only bibliographic review published about researchers in the field of dentistry and oral sciences in Nigeria. There are a few limitations with the study design. We counted each article listing authors who met the inclusion criteria such that if two authors collaborated in one paper, the count of the article was 2. This may lead to the over-estimation of the number of articles published. We were unable to control for some confounders like length of career (47) because we did not have data on employment status. However, we assumed that this would affect both genders equally and as such, it was a missing variable that could only introduce minimal bias to the study. Also, we had no access to data on leave of absence due to reasons like parental care (a factor that is likely to affect women more than men), the sex proportion of the research workforce (and so the research productivity could not be weighted per sex), and the vast cultural, geographical, political and religious diversity of Nigeria (the gender roles and values that may affect sex differences in research productivity were not controlled for). Despite these limitations, the study findings provided some insights that may influence gender defined support for oral health researchers.

First, like prior studies, we found sex differences in the productivity and impact of research productivity, impact and collaboration pattern. Unlike prior studies (48, 49) conducted in high- and upper middle-income countries (50–52), female researchers had significantly higher research productivity and impact than male researchers. A prior report had also observed no gender difference in research productivity in the field of oral and maxillofacial surgery specialty (47). This observed reversal of gender differences in dental research productivity and impact when compared with reports from higher income countries, may be related to gender roles. Men are the breadwinners in many homes in Nigeria (53). The poor economic condition of the country over the last decade or more, may have made men pay less attention to article publications. The article processing fees for manuscript publications are not supported by research institutions in Nigeria. Nigeria is also one of the countries with the lowest research funding in the world, contributing less than 0.22% of its gross domestic product to research (54).

On the other hand, collaborative research facilitates access to funds for manuscript publication. This may explain the reason why we observed more males than females involved in domestic and international collaborations. This postulation may also explain the higher female research productivity and impact wherein more female dental academia in Nigeria are engaged with publishing articles as they are not majorly saddled with the responsibility of stabilizing household income during the worsening financial crisis in the country. Female dental researchers may therefore, be investing their time better in research productivity during their work hours than the male researchers. This postulation needs to be studied further.

The postulation that considerations about personal economic benefits may affects the productivity of male researchers in Nigeria, has a number of interpretations and implications. One, we hypothesize that when the research financial environment is favorable, male dominance in the oral health research field in Nigeria becomes magnified. Two, when family duties, the community and national economy affect the financial security of men, research is given less priority. There are prior indications that the political, and therefore the economic stability of countries, affect oral health research productivity (29). Thus, the competency of male oral health researchers in Nigeria may be better than that of female when there is political and economic stability. This hypothesis implies that our study findings should be treated with caution and the findings contextualized for meaningful interpretation.

Second, the observed significantly higher percentage of females listed as first authors and a significantly higher percentage of males listed as last authors may indicate more males are senior career researchers, and thus, support our earlier hypothesis. First authorship connotes the researcher whose work underlies the article as a whole (55), whereas the last authorship connotes the researcher whose work made the study possible, or the driving financial and intellectual force of the research (55–57). Prior comprehensive studies had shown low odds of female being last author in every continent, country, journal category and journal studied (48). The current study corroborates prior study findings.

It is also possible that the results indicating that significantly more female dental researchers had WoS publications per author, first authorship, and more citation per publication is suggestive of a progress being made in Nigeria with respect to gender equality in dentistry and oral research. Albeit, this is not a reflection of the gender equality status in the country. Nigeria has a low gender equality ranking status of 0.33% by the World Bank in 2020 (58). Prior comprehensive studies of dental publications and general dental literature showed poorer female productivity irrespective of dental disciplines, countries and across first and last authorship (49, 59). There are, however, reports of an increase in the number of females applying to, studying, qualifying, and practicing as dentists in the global North over the past half a century due to educational and practice systems that provide both males and females with equal opportunities (60–62). In Nigeria, the number of females in dental institutions had steadily increased from 36.2% in 2003, to 42.5% in 2013 (63). However, an increase in opportunities for females to have access to medical and dental education does not translate to improved female academic productivity (64, 65). Also, an increase in the number of female first authorship does not translate to increase in female senior researcher with time (66, 67). Further studies are needed to understand the study finding.

Third, the observation that male researchers may be the dominant senior researchers due to their listing as last author and more involvement with collaborative research may have implications for mentorship. Early female career researchers may face challenges with access to women as mentors. This is further corroborated by evidence that indicates that men are less able to challenge women mentees to do and experience things they might otherwise neglect or even actively avoid (68). Female mentors are better able to play this role (68). Sartori et al. demonstrated that *having a woman as the last author increased the presence of women in the first author position in scientific dental articles by 16%* (59). Our study finding on the correlation between first authorship and last female authorship and non-significant correlation between first authorship and last male authorship supports this postulation. Addressing this significant gender disparity in first and last authorships of dental researchers might be helpful to accelerate the burgeoning move toward gender equality in all aspects of dentistry and oral science research in Nigeria.

## Conclusion

Overall, the observed gender profile implies a positive step towards gender equality in dentistry and oral sciences research productivity in Nigeria. The current gender status suggests that female dental researchers may have relatively high research productivity and impact. The significant gender disparity in first and last authorship suggests the need for caution in the interpretations of the results as there is the possibility of socioeconomic and cultural gender nuances influencing the observed study outcomes. Future studies are recommended to explore the study findings.

## Data Availability

The original contributions presented in the study are included in the article/Supplementary Material. Further inquiries can be directed to the corresponding author.
